# Prediction of esophagogastroduodenoscopy therapeutic usefulness for in-ICU suspected upper gastrointestinal bleeding: the SUGIBI score study

**DOI:** 10.1186/s13613-024-01250-0

**Published:** 2024-02-15

**Authors:** Victor Penaud, Thibault Vieille, Tomas Urbina, Vincent Bonny, Paul Gabarre, Louai Missri, Maxime Gasperment, Jean-Luc Baudel, Nicolas Carbonell, Alexandra Beurton, Sayma Chaibi, Aurélia Retbi, Muriel Fartoukh, Gaël Piton, Bertrand Guidet, Eric Maury, Hafid Ait-Oufella, Jérémie Joffre

**Affiliations:** 1 Medical Intensive Care Unit, Saint Antoine University Hospital, APHP, Sorbonne University, 75012 Paris, France; 2 Intensive Care Unit, Besançon University Hospital, 25000 Besançon, France; 3 Gastroenterology Department, AP-HP, Hôpital Saint-Antoine, Sorbonne University, 75012 Paris, France; 4 Intensive Care Unit, Tenon University Hospital, APHP, Sorbonne University, 75020 Paris, France; 5 Département d’Information Médicale, Hôpital Saint Antoine, Assistance Publique-Hôpitaux de Paris, Sorbonne University, Paris, France; 6 Pierre Louis Institute of Epidemiology and Public Health, Inserm U1136, Sorbonne University, Paris, France; 7 Paris Cardiovascular Research Center, Inserm U970, Paris Center University, Paris, France; 8 Centre de Recherche Saint-Antoine, Inserm UMRS-938, Sorbonne University, Paris, France

**Keywords:** Gastrointestinal bleeding, Esophagogastroduodenoscopy, Hemostasis, Endotherapy, «Stress» ulcer

## Abstract

**Background:**

Suspected upper gastrointestinal bleeding (SUGIB) is a common issue during ICU stay. In the absence of specific guidelines on the indication and timing of esophagogastroduodenoscopy (EGD), there is substantial variability in EGD indication depending on accessibility and clinical presentation. This study aimed to investigate factors associated with the need for per-EGD hemostatic therapy and to create a score predicting therapeutic benefit of emergency bedside EGD in ICU patients with SUGIB.

**Methods:**

We conducted a retrospective study in our ICU to identify factors associated with the need for hemostatic procedure during EGD performed for SUGIB. From this observational cohort, we derived a score predicting the need for hemostasis during EGD, the SUGIBI score. This score was subsequently validated in a retrospective multicenter cohort.

**Results:**

Two hundred fifty-five patients not primarily admitted for GI bleeding who underwent a bedside EGD for SUGIB during their ICU stay were analyzed. The preeminent EGD indication were anemia (79%), melena (19%), shock (14%), and hematemesis (13%). EGD was normal in 24.7% of cases, while primary lesions reported were ulcers (23.1%), esophagitis (18.8%), and gastritis (12.5%). Only 12.9% of patients underwent hemostatic endotherapy during EGD. A SUGIBI score < 4 had a negative predictive value of 95% (91–99) for hemostatic endotherapy [AUC of 0.81; 0.75–0.91 (*p* < 0.0001)]. The SUGIBI score for predicting the need for an EGD-guided hemostatic procedure was next validated in a multicenter cohort with an AUC of 0.75 (0.66–0.85) (*p* < 0.0001), a score < 4 having a negative predictive value of 95% (92–97).

**Conclusions:**

Our study shows that the therapeutic usefulness of bedside emergency EGD for SUGIB in critically ill patients is limited to a minority of patients. The SUGIBI score should help clinicians stratify the probability of a therapeutic EGD.

**Supplementary Information:**

The online version contains supplementary material available at 10.1186/s13613-024-01250-0.

## Background

Suspicion of upper gastrointestinal bleeding (UGIB) is a common situation in critically ill patients during ICU stay [1–4]. Bleeding may be overt, characterized by exteriorized hematemesis, melena, or hematochezia, and lead to hemodynamic instability [5]. However, UGIB is often suspected in other nonspecific situations, without any exteriorized bleeding, as many critically ill patients experience progressive decrease in hemoglobin levels and require RBC transfusion during ICU stay. Besides multifactorial «stress» ulceration associated with critical illness, UGIB in ICU can be of multiple origins, such as esophagitis, gastritis, esophageal varices (EV), or any other lesion responsible for UGIB [5–9]. More rarely, the bleeding originates from the lower gastrointestinal tract.

While the management of acute gastrointestinal bleeding in patients presenting to the emergency department is well established with international guidelines [10, 11] there is no consensus regarding the management of suspected UGIB (SUGIB) occurring during an ICU stay. Despite some heterogeneity in local practices and access to endoscopy procedures, bedside esophagogastroduodenoscopy (EGD) is frequently performed in ICU patients [12]. It serves both a diagnostic and potential therapeutic role, with hemostatic procedure such as hemostatic clips, vasoconstrictor injection, or EV ligation if required. However, certain superficial mucosal lesions such as esophagitis, nasogastric tube (NGT)-associated ulcerations, or gastritis usually do not require endoscopic hemostatic treatment. Currently there is no available data on the incidence of hemostatic endotherapy during EGD performed for SUGIB in critically ill patients. On the other hand, performing an EGD on a critical patient is a procedure with potential risks [13–15], and substantial human and material costs [16]. Therefore, in case of high probability of no EGD hemostatic procedure, the EGD benefits must be weighed against its inherent costs and risks.

Our study aimed to first describe the results of EGD performed in the ICU for SUGIB during ICU stay in patients admitted for another reason than acute UGIB, in terms of diagnostic and therapeutic performances. Second, we analyzed predicting factors associated with an EGD hemostasis and proposed a simple stratification score based on clinical items allowing intensivists to predict the probability of therapeutic EGD hemostasis and subsequently help reconsider performing an EGD. The performances of this score were then evaluated in a multicentric validation cohort.

## Methods

### Patients and data collection

For the derivation cohort, we conducted a retrospective, monocentric study in an 18-bed ICU in a university hospital. Our ICU is a tertiary center for gastrointestinal bleeding (GIB) in Paris, France, and has 24/7 EGD access. Using the administrative hospital database (PMSI), we screened all patients who underwent a bedside EGD in our ICU between January 2015 and October 2021. For the validation cohort, we used data from 3 teaching centers’ ICUs: Besançon and Tenon university hospitals, from January 2015 to December 2022, and Saint-Antoine hospital (same as the derivation cohort) with patients from November 2021 to December 2022. We excluded patients admitted to the ICU for upper or lower GIB, hemorrhagic shock, or in whom EGD was performed for any other reason than suspicion of upper GIB (SUGIB). SUGIB occurring less than 10 days after an endoscopic retrograde cholangiopancreatography (ERCP) or scheduled EGD were also excluded. In the case of multiple EGDs during ICU stay, only the first was analyzed for the score derivation and validation. The flowchart is provided in Additional file 1: Figure S1. Demographic, clinical, biological, and endoscopic data were collected, as well as outcomes (in-ICU and in-hospital mortality and length of stay). Patients received written information that data extracted from their medical charts could be used for research. According to French legislation, the database was anonymized and registered by the CNIL (N°2226507), and the project received approval from the French intensive care society ethical committee (CE SRLF 23-049).

### Local practice for stress ulcer prevention and EGD procedure

According to our ICU policy (derivation cohort), no systematic ulcer prevention is given to patients [17, 18]. We use proton-pump inhibitors (PPIs) only in patients with two or more risk factors of «stress» ulcer amongst the following: previous history of ulcer, antiplatelet drugs, curative anticoagulation, or acute kidney injury (AKI) or in case of multiorgan failure. All patients with SUGIB receive PPIs at a dose of 8 mg/h before EGD, and Octreotide (25–50 µg/h) is also given only in patients with known cirrhosis or suspected portal hypertension. An erythromycin infusion (250 mg over 30 min) is performed 30–60 min before endoscopy if no contraindication. According to our local policy, all bedside in-ICU EGDs are performed under general anesthesia and after endotracheal intubation (ETI) if not already intubated for another reason [19–22], by an experienced gastroenterologist.

### Statistics

Results are reported as means (± SD) or median (IQR) for continuous variables and as percentages for qualitative variables. To assess associations between patient characteristics and EGD-guided hemostatic procedures, we first performed univariate analyses based on the Mann–Whitney or chi-square test as appropriate. To identify independent predictors of EGD-guided hemostatic procedure, a multivariable logistic regression model included variables with *p*-values less than 0.05 by univariate analysis. The model's goodness of fit was assessed using the Hosmer–Lemeshow test and the discrimination by the area under the receiver operating characteristic curve (ROC AUC). All tests were two-sided, and *p*-values less than 0.05 were considered statistically significant. Statistics were performed using R (https://www.R-project.org/) software, and graphical representations using GraphPad Prism 9.00 (GraphPad Software Inc.®). The Transparent Reporting of a multivariable prediction model for individual prognosis or diagnosis (TRIPOD) guidelines were applied [23].

## Results

### Patient characteristics at baseline and during ICU stay—derivation cohort

We identified 431 ICU patients who underwent bedside EGD during the inclusion period. One hundred seventy-six patients were excluded, primarily because admitted for acute GIB or hemorrhagic shock (*n* = 133) or other indication than SUGIB (*n* = 37). Two hundred and fifty-five patients were included in our study (the flowchart is provided in Additional file 1: Figure S1), mainly men (65.9%), with a mean age of 64 ± 15 years. 11.9% had a previous history of ulcers, and 17.6% had known cirrhosis. Before ICU admission, 27.8% were on antiplatelet therapy, 18.5% had curative anticoagulants, and 32.7% had PPIs. The main reason for ICU admission was respiratory failure (35.3%), followed by sepsis/septic shock (22.4%) and neurological disorders (16.5%). The mean SAPS II at admission was 51 ± 19. During ICU stay, 66.7% had sepsis, 75% were mechanically ventilated (disregarding intubation solely for EGD), 56.4% required vasopressors, and 34.1% received renal replacement therapy (RRT) (hemodialysis only). The median ICU length of stay was 12 [6–23] days, and ICU mortality was 27.4%. Table 1 summarizes patient characteristics at baseline and during ICU stay.

**Table 1:** Patient characteristics at baseline and during ICU stay—derivation cohort

Baseline patient characteristics (*n* = 255)	
Age (years, mean ± SD)	64 ± 15
Male, *n* (%)	168 (65.9)
Medical history, *n* (%)	
Ulcer	29 (11.4)
Cirrhosis	45 (17.6)
Esophageal varices	5 (2)
Cardiovascular comorbidity	134 (52.5)
CKD	33 (12.9)
Chronic RRT	7 (2.7)
Cancer/hematological malignancies	81 (31.8)
Digestive surgery	64 (25.1)
Diabetes mellitus	51 (20)
COPD/asthma	31 (12.2)
HIV	6 (2.4)
Medication, *n* (%)	
Antiplatelet drugs	71 (27.8)
Anticoagulants	47 (18.5)
NSAIDs	14 (5.5)
Steroids	32 (12.6)
Chemotherapy	29 (11.4)
PPIs	83 (32.7)
ICU admission cause, *n* (%)	
Sepsis/septic shock	57 (22.4)
Cardiac arrest/cardiogenic shock	13 (5.1)
Respiratory	90 (35.3)
Neurologic	42 (16.5)
Metabolic	26 (10.2)
Others	27 (10.6)
**Admission SAPSII (mean ± SD)**	51 ± 19
ICU stay characteristics and treatment, *n* (%)	
Sepsis	170 (66.7)
Invasive mechanical ventilation	191 (74.9)
RRT	87 (34.1)
Vasopressors	144 (56.4)
Anticoagulant	71 (27.8)
Steroids	54 (21.2)
Antiplatelets	52 (20.4)
Outcome	
ICU LOS (days, mean ± SD)	17.5 ± 17.6
Hospital LOS (days, mean ± SD)	35 ± 34.5
In-ICU mortality, *n* (%)	70 (27.4)
In-hospital mortality, *n* (%)	87 (34.1)

*SD* standard deviation, *CKD* chronic kidney disease, *RRT* renal replacement therapy, *COPD* chronic obstructive pulmonary disease, *NSAID* non-steroidal anti-inflammatory drug, *PPI* proton pump inhibitor, *ICU* intensive care unit, *SAPSII* simplified acute physiology score II, *LOS* length of stay

### EGD indication, procedure, and results

**Fig. 1:**
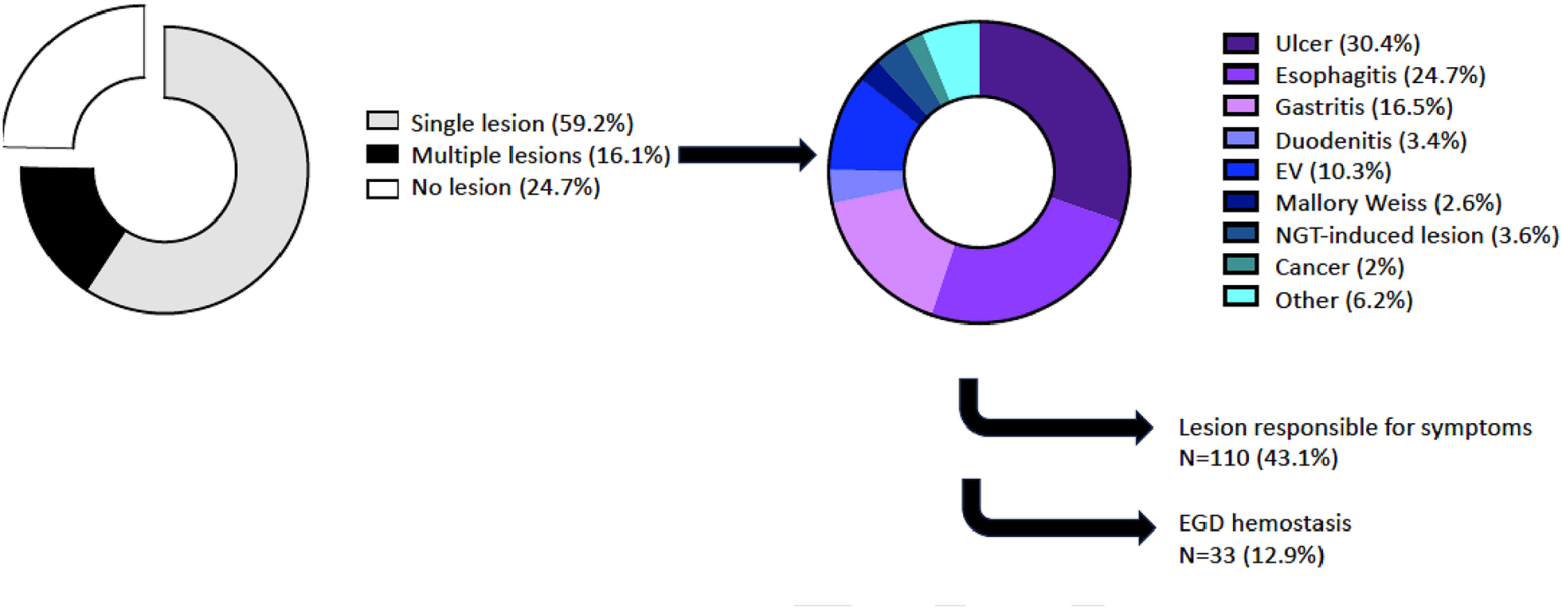
EGD findings and distribution. *EGD* esophagogastroduodenoscopy, *EV* esophageal varices, *NGT* nasogastric tube, *UGIB* upper gastrointestinal bleeding

EGD for SUGIB was performed 8.3 ± 13 days after ICU admission. The procedure was primarily motivated by anemia in 79.6% of cases, followed by melena (19.2%), hemodynamic instability (14.5%), and hematemesis/blood in the nasogastric tube (NGT) (13.5%). In 61 patients, ETI was performed specifically for the procedure. Notably, no complication related to airway management was observed in patient requiring ETI. EGD was reported as normal in 24.7% of cases, whereas a single lesion was identified in 59.2% and multiple lesions in 16.1% of cases. The main EGD findings were ulcers (23.1%), esophagitis (18.8%), gastritis (12.5%), and EV (7.8%) (Table 2 and Fig. 1). Ultimately, an endoscopically identified lesion was considered responsible for UGIB in 43.1% of cases. Per-EGD endotherapy was performed in 33 patients (12.9%), using mainly hemostatic clipping (*n* = 19, 7.5% of patients, 58% of hemostatic procedures), epinephrine local instillation (*n* = 16, 6.3% of patients, 48% of hemostatic procedures) or EV ligation (*n* = 14, 5.5% of patients, 42% of hemostatic procedures) (Table 2). Twenty-four (9.4%) patients required a second EGD, 9 (3.5%) had a colonoscopy or rectosigmoidoscopy, 5 (2%) had an arterial embolization and 5 (2%) hemostatic surgery. After EGD, PPI treatment was recommended in 70.2% of cases. During ICU stay, patients received 2.2 ± 3.5 RBC packs, 0.5 ± 2 platelet units/10 kg, and 0.4 ± 1.7 units of fresh frozen plasma (FFP) (Additional file 1: Table S1). Biological parameters on EGD day are reported in Additional file 1: Table S2.

**Table 2:** EGD indication, procedure, and results

	EGD (*n* = 255)
**Time from admission (days, mean ± SD)**	**8.3 ± 13**
**EGD indication**, *n* (%)*	
Anemia	203 (79.6)
Melena	49 (19.2)
Hemodynamic instability	37 (14.5)
Hematemesis/blood in NGT	35 (13.7)
Hematochezia	35 (13.7)
**Intubation solely for EGD, *n* (%)**	**61 (23.9)**
**EGD results**, *n* (%)	
Normal	62 (24.3)
Ulcer	59 (23.1)
EV	20 (7.8)
Esophagitis	48 (18.8)
Mallory–Weiss	5 (2)
Gastritis	32 (12.5)
NGT-induced lesion	7 (2.7)
Other	12 (4.7)
Multiple lesions	41 (16.1)
Lesion considered responsible for GI bleeding	110 (43.1)
**Hemostatic procedure**, *n* (%)**	**33 (12.9)**
Hemostatic clip	19 (58)
Epinephrine instillation	16 (48)
EV ligature	14 (42)
Other (Hemospray™/Gold probe™/APC)	5 (15)

*EGD* esophagogastroduodenoscopy, *NGT* nasogastric tube, *EV* esophageal varices, *EBO* endobrachyesophagus, *GI* gastrointestinal, *APC* Argon plasma coagulation. *Some patients had EGD for multiple indications. **Some patients received multiple means of hemostasis during EGD

### Factors associated with hemostatic endotherapy procedures

**Fig. 2:**
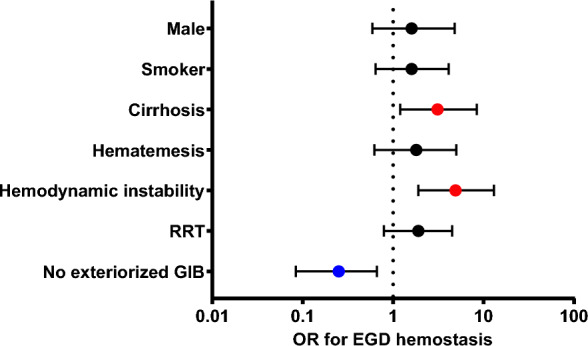
Multivariate analysis: predictors of hemostatic endotherapy. *EGD* esophagogastroduodenoscopy, *OR* odds ratio, *RRT* renal replacement therapy, *GIB* gastro intestinal bleeding. The dots represent the odds ratio; colored dots are used when the 95% confidence interval for the OR does not include 1. The line through each dot corresponds to the 95% confidence interval. Variables with *p* < 0.05 entered in the maximal model for multivariate analysis. Goodness of fit (Hosmer–Lemeshow statistic) *p* = 0.4, Turj *r* squared = 0.23. Calibration (AUC-ROC) 0.84; *p*-value < 0.001

Next, we analyzed the differences between patients with or without EGD-guided hemostatic procedures. In univariate analysis, we observed that patients requiring EGD hemostasis were more often men (81.8% vs. 63.5%, *p* = 0.04) and more frequently had a history of cirrhosis (36.4% vs. 14.9%, *p* = 0.006), smoking (66.7% vs. 46%, *p* = 0.04), and daily alcohol intake (57.6% vs. 37.4%, *p* = 0.04). Patients who required hemostasis per EGD had more frequent RRT (48.5% vs. 32%, *p* = 0.008). Regarding EGD indication, hematemesis/blood in NGT (36.4% vs. 10.4%, *p* < 0.0001), hematochezia (30.3% vs. 11.3%, *p* = 0.003), and shock (42.4% vs. 10.4%, *p* < 0.0001), were more frequent in the group with per EGD hemostasis. Conversely, absence of blood exteriorization was three times more frequent in the group without therapeutic hemostasis (21.2% vs. 64%, *p* < 0.0001). Interestingly, the type of lesion was similar in both groups except for EV (24.2% vs. 5.4%, *p* = 0.0002). Biological parameters on which the EGD was performed were similar in both groups except for platelet count and prothrombin time, which were significantly lower in patients requiring EGD hemostasis (Additional file 1: Table S2). Ultimately, patients with per-EGD hemostasis received PPI medication, RBC, platelet, and FFP transfusions more often. Nevertheless, both groups had similar ICU mortality and length of stay (Additional file 1: Table S3). In multivariate analysis, history of cirrhosis (OR 3.1; 1.2–8.4, *p* = 0.02) and hemodynamic instability indicating EGD (OR 4.9; 1.9–13, *p* = 0.0009) were significantly associated with hemostatic endotherapy. Conversely, no exteriorized bleeding was negatively associated with hemostatic endotherapy (OR 0.25; 0.09–0.66, *p* = 0.007) (Fig. 2 and Additional file 1: Table S4).

### Derivation of the *SUGIBI* score

**Fig. 3:**
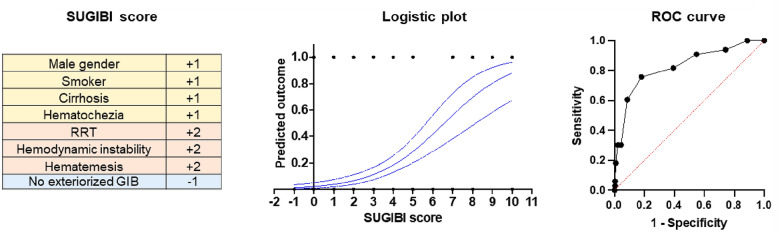
The SUGIBI score. The SUGIBI score with a threshold < 4 has a negative predictive value of 95% (0.91 to 0.99). Area under the ROC curve: 00.81; 0.75–0.91 (*p* < 0.0001). *EGD* esophagogastroduodenoscopy, *RRT* renal replacement therapy, *GIB* gastrointestinal bleeding, *ROC* receiver operating curve

Based on the differences between patients who required EGD hemostasis and those who did not, and after best subsets logistic regression analyses, we build the *SUspected GIB in Icu* score (Additional file 1: Figure S2). In the SUGIBI score, male gender, smoking, history of cirrhosis and hematochezia were attributed one point. RRT, hemodynamic instability (with no patent alternative etiology) and/or hematemesis indicating EGD were attributed two points. No external bleeding was associated with one negative point. This score had an AUC of 0.81; 0.75–0.91 (*p* < 0.0001) for predicting the need for an EGD-guided hemostatic procedure. More interestingly, a SUGIBI score < 4 had a negative predictive value for the need for hemostatic endotherapy of 95% (91–99) (Fig. 3). The performance of the Blatchford bleeding score to predict need for endotherapy (AUC: 0.64; 0.53–0.74; *p* = 0.012) in this ICU context, was significantly lower compared to the SUGIBI score (*p* < 0.0001) (Additional file 1: Figure S3).

### Performances of the SUGIBI score in the validation cohort

The flowcharts and patient characteristics of the validation cohort are provided in Additional file 1: Figure S4 and Additional file 1: Table S5, respectively. Three hundred and thirty-four patients from 3 centers were used as the SUGIBI score validation cohort. They were mainly men (70.4%) with an age of 62.4 ± 15 years, and were of similar severity compared to the derivation cohort (admission SAPS II of 50 ± 19, ICU mortality of 29.6%). Amongst the 334 EGD performed for SUGIB, 108 (32%) were described as normal, 154 (46.1%) identified a lesion considered responsible for the bleeding, and 32 (9.6%) had a hemostatic endotherapy procedure. Forty-six patients (13.8%) were intubated (or re-intubated) solely for the EGD, with three cardiac arrests occurring during these procedures. In the validation cohort, the SUGIBI score had an AUC of 0.75 (0.66–0.85) (*p* < 0.0001) for predicting the need for an EGD-guided hemostatic procedure. A score < 4 had a negative predictive value of 95% (92–97).

## Discussion

This study reports two main findings. First, we found that EGD performed during ICU stay in critically ill patients for SUGIB identified upper GI lesions in 75% of cases, of which 43.5% were deemed responsible for the bleeding, and ruled out the diagnosis of UGIB in 25% of cases in the derivation cohort, and in 29% of the overall population (derivation and validation cohorts). Therefore, EGD is a valuable diagnostic tool and may help guide PPI or octreotide continuation or withdrawal [24, 25]. Interestingly, critically ill patients with UGIB that occurred during the ICU stay had similar sources of bleeding than patients admitted to the hospital specifically for GIB, as previously reported [5, 26]. However, in the overall population of our study (derivation and validation cohorts), a hemostatic procedure was performed in only 11%, of cases and, therefore, that EGD had no direct therapeutic value in 89% of patients.

The second finding of this study is that patients requiring an EGD-guided hemostatic procedure are significantly different from those who do not. Therefore, we propose a simple score based on clinical criteria, able to identify patients with a low probability of needing EGD hemostasis, with a negative predictive value > 90%. In 2000, Blatchford et al. published the Blatchford score to stratify the probability of need for treatment in patients admitted to the ER for UGIB, and since then other scores have been proposed [27, 28]. Our study reveals that similar items are valid in the ICU SUGIB context, such as hemodynamic instability, exteriorized bleeding, and liver disease (cirrhosis). Nevertheless, some criteria such as tachycardia, decrease in hemoglobin, or increased urea, are ineffective in the context of critically ill patients because of multiple confounding factors related to the high prevalence of multiorgan failure and numerous putative causes of anemia [29] and tachycardia [30], highlighting the interest of a specific score for ICU patients. In addition, our study unveils some unexpected factors associated with the need for an EGD hemostasis, such as male gender and smoking status. This could be explained by the pathophysiology of «stress» ulcers, whose primary driver is mucosal ischemia and is, consequently, more frequent in high cardiovascular-risk patients [28, 31].

Hence, we propose that patients with a SUGIBI score < 4 might have a delayed EGD, assuming PPI treatment until the EGD. Indeed, if we applied the SUGIBI score to our cohort, 459 EGDs without hemostatic intervention could have been avoided, representing a substantial gain in caregiver time and spared costs. Moreover, in our series, general anesthesia and intubations performed solely for the EGD would have been avoided in 74 patients. Although no intubation failure or significant complication was reported in our derivation cohort, it is well established that such airway management procedures in full stomach conditions are at risk of complications [32–34], as illustrated in the validation cohort where three cardiac arrests related to the procedure were reported. In many centers, EGD is not available 24/7, and sometimes such a procedure requires transferring the patients to another hospital. Therefore, the SUGIBI score may help to better stratify the patients and reduce endotherapy-free EGDs performed for SUGIB by reconsidering the need for emergency EGD in case of a score < 4. Conversely, in the case of a SUGIBI score ≥ 4, we believe that EGD must be performed without delay, given the substantial probability of hemostatic endotherapy.

This study has several limitations. First, the monocenter derivation cohort was performed in a high volume/highly experienced GIB center with 24/7 EGD availability. Therefore, our local practice of having broad EGD indications cannot be generalized. Nevertheless, such design was decided on purpose to overcome indication bias. Surprisingly, we observed that in the 2 other centers the percentage of endotherapy was identical to our center, suggesting that despite differences in recruitment and practice, the proportion of patients requiring endotherapy for EGD is consistently around 10%. Second, our local practice is to perform any bedside EGD under general anesthesia, which often leads to (re)intubating the patient. This is an at-risk procedure, with a 10 to 15% complication rate [35–37]. As no complication was reported in our derivation cohort and only 3 in the validation cohort, we suspect an underreporting bias. Moreover, our conclusion on reducing intubation numbers using the SUGIBI score could not be extrapolated to centers not systematically performing intubation for EGD. Last, this score is a dynamic index, as the clinical status of a patient with active GIB is unstable and must be reassessed daily before ruling out the need for a putatively therapeutic EGD.

## Conclusion

Our study shows that the therapeutic usefulness of bedside emergency EGD for SUGIB in critically ill patients is limited to a minority of patients. The SUGIBI score could help clinicians stratify the probability of a therapeutic EGD. In case of a SUGIBI score < 4, EGD might be postponed or reconsidered whereas in patients with a SUGIBI score ≥ 4, EGD should be performed without delay.

### Supplementary Information

**Additional file 1.** Supplemental Tables 1-5 and Supplemental figures 1-4.

## Data Availability

The full dataset is available from the corresponding author, on request.

## Abbreviations

AKI: Acute kidney injury
APC: Argon plasma coagulation
AUC: Area under the curve
CKD: Chronic kidney disease
CNIL: Commission nationale informatique et liberté
COPD: Chronic obstructive pulmonary disease
EBO: Endobrachyesophagus
EGD: Esophagogastroduodenoscopy
ETI: Endotracheal intubation
EV: Esophagial varices
FFP: Fresh frozen plasma
ICU: Intensive care unit
IQR: Interquartile range
LOS: Length of stay.
NGT: Nasogastric tube
NSAID: Non-steroidal anti-inflammatory drugs
PMSI: Programme de médicalisation des systèmes d’information
PPI: Proton pump inhibitor
RBC: Red blood cells
ROC: Receiving operating curve
RRT: Renal replacement therapy
SAPSII: Simplified acute physiology score II
SD: Standard deviation
SUGIB: Suspected upper gastrointestinal bleeding
UGE: Upper gastrointestinal endoscopy
UGIB: Upper gastrointestinal bleeding
