# Supplementary material for: Prediction of esophagogastroduodenoscopy therapeutic usefulness for in-ICU suspected upper gastrointestinal bleeding: the SUGIBI score study
Source: Ann Intensive Care. 2024 Feb 15;14:28. doi: 10.1186/s13613-024-01250-0 (PMC10869326; doi:10.1186/s13613-024-01250-0)
Supplement: Supplementary file 1 — Additional file 1. Supplemental Tables 1-5 and Supplemental figures 1-4. [file 13613_2024_1250_MOESM1_ESM.docx]

**Supplemental Figure 1: Study flow chart – Derivation cohort**


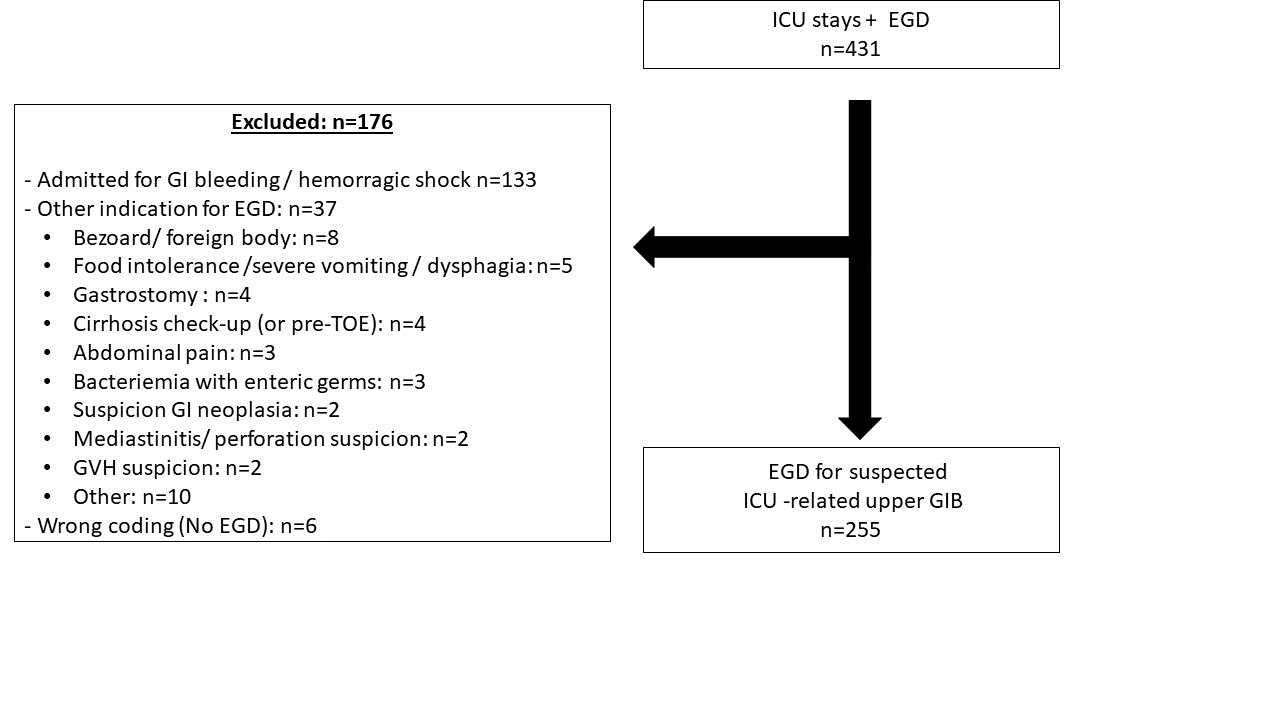


**Supplemental figure 1 abbreviations:** ICU, intensive care unit ; EGD, **esophagogastroduodenoscopy**; GI, gastrointestinal; TOE, transoesophageal echocardiography; GVH, graft versus host; GIB, gastrointestinal bleeding.

**Supplemental table 1: Post-EGD medical treatment and additional therapy**

| Medical treatment |  |
| --- | --- |
| PPI, n (%) | 179 (70.2) |
| RBC transfusion, n (%) | 130 (51) |
| RBC (unit, mean± SD) | 2.2 ± 3.5 |
| Platelet transfusion, n (%) | 37 (14.5) |
| Platelet (unit/10Kg, mean± SD) | 0.5 ± 2 |
| FFP transfusion, n (%) | 26 (10.2) |
| FFP (unit, mean± SD) | 0.4 ± 1.7 |
| Additional therapy, n (%) |  |
| Second EGD, n (%) | 24 (9.4) |
| Embolization, n (%) | 5 (2) |
| Surgery, n (%) | 3 (1.3) |
| Colonoscopy/rectosigmoidoscopy, n (%) | 9 (3.5) |

**Supplemental table 1 abbreviations:** EGD, **esophagogastroduodenoscopy**; PPI, Proton pump inhibitors; RBC, red blood cells; FFP, fresh frozen plasma; SD, standard deviation.

**Supplemental table 2: Biologicals values on the EGD day**

| Biologicals (mean ± SD) |  | Univariate analysis | | |
| --- | --- | --- | --- | --- |
|  | All cohort  (n=255) | No endotherapy (n=222) | Endotherapy (n=33) | *P* (Mann-Whitney) |
| Hemoglobin (g/dL) (n=148) | 8.1 ± 1.9 | 8.1 ± 1.9 | 7 ± 1.4 | 0.09 |
| Hematocrit (%)(n=148) | 25 ± 6.8 | 25 ± 6.9 | 21 ± 4.8 | 0.11 |
| Platelets (10^6/mL) (n=131) | 185 ± 148 | 186 ± 151 | 173 ± 119 | 0.97 |
| Urea (mmol/L) (n=129) | 20 ± 17 | 20 ± 17 | 23 ± 13 | 0.17 |
| Creatinine (µmol/L) (n=129) | 212 ± 207 | 207 ± 208 | 275 ± 198 | 0.22 |
| HC03^-^ (mmol/L) (n=125) | 23 ± 6.5 | 23 ± 6.7 | 23 ± 4.4 | 0.83 |
| PT (%) (n=125) | 68 ± 20 | 67 ± 21 | 69 ± 12 | 0.79 |
| Lactate (mmol/L) (n=102) | 2.2 ± 3 | 2.2 ± 3.1 | 2.2 ± 1.9 | 0.81 |

**Supplemental table 2 abbreviations:** SD, standard deviation; PT, Prothrombin time.

**Supplemental table 3: Univariate analysis of factors associated with EGD hemostasis.**

|  | No endotherapy (n=222) | Endotherapy (n=33) | *P*  (*Mann-Whitney or chi square*) |
| --- | --- | --- | --- |
| Age (years. mean± SD) | 65 ± 15 | 63 ± 15 | 0.41 |
| Male, n (%) | 141 (63.5) | 27 (81.8) | 0.04 |
| Medical history, n (%) |  |  |  |
| Ulcer | 27 (12.2) | 2 (6.1) | 0.39 |
| Cirrhosis | 33 (14.9) | 12 (36.4) | 0.006 |
| Cardiovascular | 116 (52.3) | 18 (58.1) | 0.57 |
| CKD | 28 (12.6) | 5 (15.2) | 0.78 |
| Cancer/hematological malignances | 72 (32.4) | 9 (27.3) | 0.69 |
| Digestive surgery | 57 (25.5) | 7 (21.2) | 0.67 |
| Diabetes mellitus | 43 (19.4) | 8 (24.3) | 0.49 |
| COPD/asthma | 26 (11.7) | 5 (15.2) | 0.57 |
| HIV | 6 (4.9) | 0 (0) | 0.34 |
| Current smoker | 102 (46) | 22 (66.7) | 0.04 |
| Medication, n (%) |  |  |  |
| Antiplatelet | 64 (28.9) | 7 (21.2) | 0.41 |
| Anticoagulant | 43 (19.4) | 4 (12.1) | 0.47 |
| NSAID | 11 (5) | 3 (9.1) | 0.4 |
| Steroids | 28 (12.6) | 4 (12.1) | 0.99 |
| Chemotherapy | 25 (11.3) | 4 (12.1) | 0.78 |
| PPI | 72 (32.4) | 11 (33.3) | 0.99 |
| ICU admission cause, n (%) |  |  |  |
| Sepsis/septic shock | 45 (20.3) | 12 (36.4) | 0.61 |
| Cardiac arrest/cardiogenic shock | 13 (5.9) | 0 (0) |  |
| Respiratory | 81 (36.5) | 9 (27.3) |  |
| Neurologic | 39 (17.6) | 3 (9.1) |  |
| Metabolic | 22 (9.9) | 4 (12.1) |  |
| Others | 22 (9.9) | 5 (15.2) |  |
| Admission SAPSII (mean ± SD) | 51 ± 20 | 50 ± 16 | 0.94 |
| ICU stay characteristics and treatment |  |  |  |
| Sepsis | 145 (65.3) | 25 (75.8) | 0.32 |
| Invasive mechanical ventilation | 164 (73.9) | 28 (84.9) | 0.2 |
| RRT | 71 (32) | 16 (48.5) | 0.008 |
| Vasopressors | 120 (54) | 24 (72.7) | 0.06 |
| Anticoagulant | 62 (27.9) | 9 (27.3) | 0.99 |
| Steroids | 47 (21.2) | 7 (21.2) | 0.99 |
| Antiplatelets | 46 (20.7) | 6 (18.2) | 0.82 |
| Delay from admission (days, mean ± SD) | 8.5 ± 13 | 6.7 ± 5.7 | 0.62 |
| EGD indication, n (%)* |  |  |  |
| Hematemesis/ blood in NGT | 23 (10.4) | 12 (36.4) | <0.0001 |
| Hematochezia | 25 (11.3) | 10 (30.3) | 0.003 |
| Melena | 39 (17.6) | 10 (30.3) | 0.08 |
| Anemia | 174 (78.4) | 29 (88.9) | 0.2 |
| Hemodynamic instability | 23 (10.4) | 14 (42.4) | <0.0001 |
| No blood exteriorization | 142 (64) | 7 (21.2) | <0.0001 |
| EGD results, n (%) |  |  |  |
| Normal | 63 (28.4) | 0 (0) | 0.0002 |
| Ulcer | 47 (21.2) | 12 (36.4) | 0.08 |
| EV | 12 (5.4) | 8 (24.2) | 0.0002 |
| Esophagitis | 41 (18.4) | 7 (21.2) | 0.7 |
| Mallory Weiss | 4 (1.8) | 1 (3) | 0.63 |
| Gastritis | 29 (13.1) | 3 (9) | 0.52 |
| NGT-induced lesion | 7 (3.1) | 0 (0) | 0.3 |
| Other | 10 (4.5) | 2 (6) | 0.7 |
| Medical treatment |  |  |  |
| PPI (n.%) | 151 (68) | 28 (84.9) | 0.05 |
| RBC transfusion, n (%) | 106 (48%) | 24 (73) | 0.009 |
| RBC (unit, mean± SD) | 2 ± 3.3 | 3.4 ± 4 | 0.007 |
| Platelet transfusion, n (%) | 29 (13.1) | 8 (24.2) | 0.11 |
| Platelet (unit/10Kg, mean± SD) | 0.5 ± 2.1 | 0.76 ± 1.8 | 0.07 |
| FFP transfusion, n (%) | 16 (7.2) | 10 (30.3) | 0.0004 |
| FFP (unit, mean± SD) | 0.3 ± 0.3 | 1.4 ± 3.1 | 0.009 |
| Outcome |  |  |  |
| ICU LOS (days. mean ± SD) | 18 ± 18 | 16 ± 12 | 0.56 |
| Hospital LOS (days. mean ± SD) | 35 ± 35 | 38 ± 34 | 0.46 |
| In-ICU mortality, n (%) | 58 (26.1) | 12 (36.4) | 0.22 |
| In-hospital mortality, n (%) | 72 (32.4) | 15 (45.5) | 0.17 |

**Supplemental table 3 abbreviations:** SD, standard deviation; CKD, chronic kidney disease; RRT, renal replacement therapy; COPD, chronic obstructive pulmonary disease, NSAID, non-steroidal anti-inflammatory drugs; PPI, proton pump inhibitors; ICU, intensive care unit; SAPSII, simplified acute physiology score II; LOS, length of stay, EGD, esophagogastroduodenoscopy; NGT, nasogastric tube; EV, esophageal varices; GI, gastrointestinal. *** Some patients had EGD for multiple indication**s.

**Supplemental table 4: Multivariate analysis: predictors of hemostatic endotherapy**

|  | OR | Lower 95% CI | Upper 95% CI | P |
| --- | --- | --- | --- | --- |
| Male | 1.6 | 0.59 | 4.8 | 0.38 |
| Smoker | 1.6 | 0.64 | 4.1 | 0.33 |
| Cirrhosis | 3.1 | 1.2 | 8.4 | 0.02 |
| Hematemesis | 1.8 | 0.62 | 5 | 0.28 |
| Hemodynamic instability | 4.9 | 1.9 | 13 | 0.0009 |
| RRT | 1.9 | 0.79 | 4.5 | 0.15 |
| No external GIB | 0.25 | 0.09 | 0.66 | 0.007 |

**Supplemental table 4 abbreviations:** EGD, esophagogastroduodenoscopy; OR, odds ratio; RRT, renal replacement therapy; GIB, gastro intestinal bleeding; CI, confidence interval.

**Supplemental Figure 2: Model performance according to several tested predictive scores**

**Supplemental Figure 2 legends: (A)** displayed the multiple combinations tested to build a predictive score of therapeutic usefulness of EGD in SUGIB and their statistical performance (B). Abbreviations: EGD: esophagogastroduodenoscopy. SUGIB, suspected upper gastrointestinal bleeding; RRT: renal replacement therapy; GIB: gastrointestinal bleeding. ROC: receiver operating curve; CI, confidence interval; NPV, negative predictive value; PPV, positive predictive value; Se, sensitivity; Spe, specificity.

**Supplemental Figure 3: Performance comparison between the SUGIBI and Glasgow-Blatchford scores to predict the therapeutic usefulness of EGD in SUGIB**

*

**Supplemental Figure 3** abbreviations: SUGIBI, suspected upper gastrointestinal bleeding in ICU; ROC: receiver operating curve; AUC, Area under the curve; CI, confidence interval; Glasgow-Blatchford* ( The GB score was modified due to the meaningless of admission cardiac frequency and blood pressure for SUGIBI: therefore in case of SUGIB + acute hemodynamic failure the score for heart rate and blood pressure was counted as 4,and 0 if no SUGIBI related hemodynamic instability).

**Supplemental Figure 4: Validation cohort - flow chart**


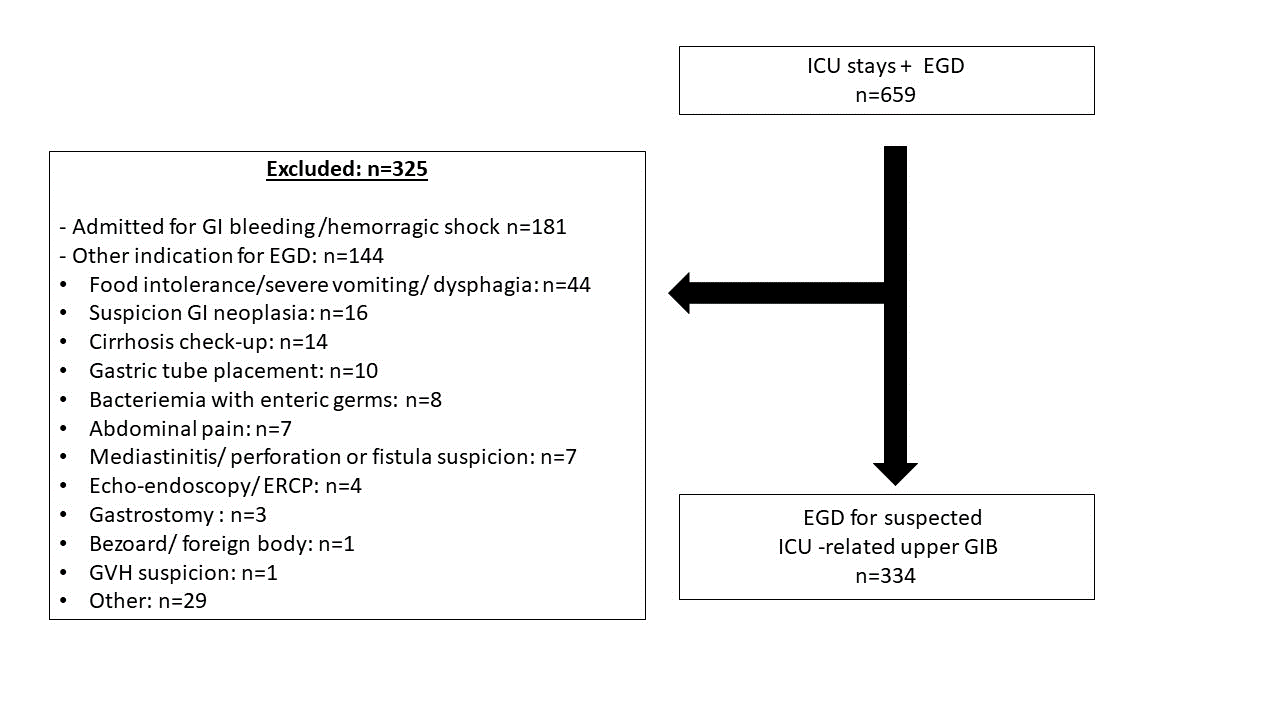


**Supplemental figure 5 abbreviations:** ICU, intensive care unit; EGD, **esophagogastroduodenoscopy**; GI, gastrointestinal; ERCP, Endoscopic retrograde cholangiopancreatography; GVH, graft versus host; GIB, gastrointestinal bleeding.

**Supplemental table 5: Validation cohort - Patients’ characteristics at baseline and during their ICU stay**

| **Baseline patient's characteristics (n=334)** | |
| --- | --- |
| Age (years, mean ± SD) | 62.4 ± 14 |
| Male, n (%) | 235 (70.4) |
| **Medical history, n (%)** |  |
| Cirrhosis | 35 (10.5) |
| Current smoker | 158 (47) |
| **ICU admission cause, n (%)** |  |
| Sepsis/ septic shock | 72 (21.6) |
| Cardiac arrest/cardiogenic shock | 29 (11.5) |
| Respiratory | 163 (48.8) |
| Neurologic | 30 (8.9) |
| Metabolic | 10 (3) |
| Others (including post-surgery) | 30 (8.9) |
| **Admission SAPSII (mean ± SD)**(n=332) | 50 ± 19.2 |
| **Time from admission for EGD (days, mean ± SD)** | 12.4 ± 15 |
| **EGD indication, n (%) *** |  |
| Anemia | 304 (91) |
| Melena | 60 (18) |
| Hemodynamic instability | 79 (23.6) |
| Hematemesis/blood in NGT | 41 (12.3) |
| Rectal bleeding/hematochezia | 31 (9.3) |
| **Intubation solely for EGD** | 46 (13.8) |
| **EGD results, n (%)** |  |
| Normal | 108 (32.3) |
| Ulcer | 67 (20) |
| EV | 9 (2.7) |
| Esophagitis | 70 (21) |
| Gastritis | 48 (14.4) |
| Other | 34 (10.1) |
| Multiple lesions | 54 (16.2) |
| Lesion considered responsible for GI bleeding | 154 (46.1) |
| **Hemostatic procedure, n (%) **** | 32 (9.6) |
| Hemostatic clip | 18 (5.4) |
| Epinephrine instillation | 12 (3.6) |
| EV ligature | 7 (2.1) |
| Other (Hemospray^TM^/ Gold probe^TM^ /APC) | 1 (0.3) |
| **Outcome** |  |
| ICU LOS (days, mean ± SD) | 26.9 ± 28.4 |
| In ICU mortality, n (%) | 99 (29.6) |
| In hospital mortality, n (%) (n=299) | 108 (36.1) |

**Supplemental table 5 abbreviations: SD, standard deviation;** ICU, intensive care unit; SAPSII, simplified acute physiology score II; LOS, length of stay; IQR, interquartile range. EGD, esophagogastroduodenoscopy; NGT, nasogastric tube; EV, esophageal varices; GI, gastrointestinal; APC, Argon plasma coagulation. *** Some patients had EGD for multiple indications. ** Some patients received multiple means of hemostasis during EGD.**
